# 3PNMF-MKL: A non-negative matrix factorization-based multiple kernel learning method for multi-modal data integration and its application to gene signature detection

**DOI:** 10.3389/fgene.2023.1095330

**Published:** 2023-02-14

**Authors:** Saurav Mallik, Anasua Sarkar, Sagnik Nath, Ujjwal Maulik, Supantha Das, Soumen Kumar Pati, Soumadip Ghosh, Zhongming Zhao

**Affiliations:** ^1^ Department of Environmental Health, Harvard T H Chan School of public Health, Boston, MA, United States; ^2^ Department of Computer Science & Engineering, Jadavpur University, Kolkata, India; ^3^ Department of Information Technology, Academy of Technology, Hooghly, West Bengal, India; ^4^ Department of Bioinformatics, Maulana Abul Kalam Azad University, Kolkata, West Bengal, India; ^5^ Department of Computer Science & Engineering, Sister Nivedita University, New Town, West Bengal, India; ^6^ Human Genetics Center, School of Public Health, The University of Texas Health Science Center at Houston, Houston, TX, United States; ^7^ Center for Precision Health, School of Biomedical Informatics, The University of Texas Health Science Center at Houston, Houston, TX, United States

**Keywords:** multi-omics, gene signature detection, feature selection, DNA methylation, matrix factorization

## Abstract

In this current era, biomedical big data handling is a challenging task. Interestingly, the integration of multi-modal data, followed by significant feature mining (gene signature detection), becomes a daunting task. Remembering this, here, we proposed a novel framework, namely, three-factor penalized, non-negative matrix factorization-based multiple kernel learning with soft margin hinge loss (3PNMF-MKL) for multi-modal data integration, followed by gene signature detection. In brief, limma, employing the empirical Bayes statistics, was initially applied to each individual molecular profile, and the statistically significant features were extracted, which was followed by the three-factor penalized non-negative matrix factorization method used for data/matrix fusion using the reduced feature sets. Multiple kernel learning models with soft margin hinge loss had been deployed to estimate average accuracy scores and the area under the curve (AUC). Gene modules had been identified by the consecutive analysis of average linkage clustering and dynamic tree cut. The best module containing the highest correlation was considered the potential gene signature. We utilized an acute myeloid leukemia cancer dataset from The Cancer Genome Atlas (TCGA) repository containing five molecular profiles. Our algorithm generated a 50-gene signature that achieved a high classification AUC score (viz., 0.827). We explored the functions of signature genes using pathway and Gene Ontology (GO) databases. Our method outperformed the state-of-the-art methods in terms of computing AUC. Furthermore, we included some comparative studies with other related methods to enhance the acceptability of our method. Finally, it can be notified that our algorithm can be applied to any multi-modal dataset for data integration, followed by gene module discovery.

## 1 Introduction

Rapid advances in biotechnology have enabled the generation of data in multiple platforms from the same or similar bio-samples. For example, The Cancer Genome Atlas (TCGA) comprehensively generated multi-omics profiles in 33 cancer types and subtypes. Therefore, it is made available to conduct an in-depth investigation into various molecular incidents at different biological stages and for specific tumor categories. The challenging task here is to develop algorithms to properly integrate these multi-omics (i.e., multi-modal) data, which will deepen our understanding of human tumorigenesis.

The integration of multi-omics profiles is a fast emerging area of the biomedical research (Imielinski et al., 2012; Mo et al., 2013; Mallik et al., 2017; Gaur et al., 2022; Ghose et al., 2022; Saeed et al., 2022). From the perspective of biology, cellular processes are based on the communication among different biomolecules (viz., mutations, epigenetic regulators, proteins, and metabolites). Molecular regulations occur in multi-layers and multi-vantage points to orchestrate complex biological events. An integrated analysis of profiles on the common set of samples from multi-omics data shows great potential to yield more biologically meaningful outcomes over an individual analysis on a single data layer. Overall, it shows a more comprehensive view and a global functional orientation of the biological system.

One of the major challenges for integration is to deal with the heterogeneity of these profiles. Profiles from various sources are often complicated to integrate or interpret together because of the inherent discrepancies. Various genomic variables can be measured and accumulated in different ways, which are also vulnerable to different kinds of noise and various confounding effects. Interestingly, these profiles show individual aspects of the biological system at different angles. The discrepancy among multi-omics data, therefore, provides an opportunity for detecting reliable and consistent signals for biological studies in a comprehensive manner. Multi-dimensional data integration and gene signature identification are among the most challenging tasks for bioinformaticians (Li et al., 2019; Mallik and Zhao, 2020; Qiu et al., 2020; Pellet et al., 2015; Serra et al., 2015). Mallik et al. (2017) proposed a scheme to recognize epigenetic biomarkers applying maximal relevance and minimal redundancy-based feature selection for multi-omics data. An approach of the integration of multi-omics data was proposed by Li et al. (2019) to identify biomarkers in the domain of cancer research. Qiu et al. (2020) suggested an approach regarding the revelation of 172 osteoporosis biomarkers by multi-omics data integration. A scheme of multi-omics data integration was presented by Pellet et al. (2015) to determine predictive molecular signatures regarding CLAD. Because specific profiles contain different characteristics/phenomena, integration of multi-view data with significant feature reduction and gene signature detection is fundamentally important. In this upcoming era, the multi-platform integration approach has been applied to accomplish various important tasks, such as signature/bio-marker detection, disease classification, and gene clustering. Prior research works in bio-marker discovery (Bandyopadhyay and Mallik, 2016; Kandimalla et al., 2022), classification (Henry et al., 2014; Maulik et al., 2015; Zhang and Kuster, 2019), and clustering (Wang and Gu, 2016) have improved the promising performance of multi-modal integration approaches. Nevertheless, the outcomes of such approaches are not always satisfactory. Zhang and Kuster (2019) represented an approach with the incorporation of proteomics data to express the significance of omics data integration with higher accuracy. Kandimalla et al. (2022) showed mRNA–miRNA regulatory network analyses to improve the approach of multi-omics data integration. In this work, we propose a novel framework, namely three-factor penalized non-negative matrix factorization-based multiple kernel learning with soft margin hinge loss (3PNMF-MKL), which applies consecutive utilization of a couple of multi-dimensional strategies: i) statistical empirical Bayes-based feature selection, ii) three-factor penalized non-negative matrix factorization, iii) multiple kernel learning with soft margin hinge loss, iv) average linkage clustering, and v) the dynamic tree cut method for multi-platform data integration and gene signature detection. For evaluation of the performance of our proposed approach, a cancer dataset from TCGA acute myeloid leukemia (LAML) containing five different profiles [gene expression, DNA methylation, exon expression, pathway activity, and copy number variation (CNV)] was used. We demonstrated that our approach is capable of multi-modal data integration, and thus, it can be applied to any kind of multi-platform datasets.

## 2 Experimental procedures

In this section, we illustrate our proposed approach for identifying Pareto-optimal gene signatures by feature clustering on a cancer multi-omics dataset. The major steps are described as follows.

### 2.1 Feature selection by the empirical Bayes test

Commonly shared features (genes/probes) and samples are chosen across all the profiles from the multi-omics cancer dataset. Specifically, probes (features) from DNA methylation arrays containing any missing values are discarded. The individual profile is normalized using the zero-mean normalization for each feature (Bandyopadhyay et al., 2013), as described in the following formula: $x_{ik}^{\prime }=\frac{x_{ik}-\mu }{\sigma }$ . Here, *μ* is the mean across the data for the feature *i* prior to normalization, and *σ* denotes standard deviation. *x*_*ik*_ and $x_{ik}^{\prime }$ signify the value of the *i*-th feature at *k*-th patient (sample) prior and after normalization, respectively. To determine statistically significant features, the empirical Bayes statistical test is applied using the package “Linear Models for Microarray and RNA-Seq Data” (Smyth, 2004; Bandyopadhyay et al., 2013), which works better on the dataset with a small sample size. The moderated t-statistic (Ritchie et al., 2015) is elaborated as follows:

$${\tilde{t}}_{pr}=\frac{1}{\sqrt{\frac{1}{m_{1}}+\frac{1}{m_{2}}}}\frac{{\hat{\beta }}_{pr}}{{\tilde{s}}_{pr}},$$ (1)

where *m*_1_ and *m*_2_ are the number of patients (cases) and that of the normal samples (controls), respectively. Here, ${\hat{\beta }}_{pr}$ signifies the contrast estimator for the feature *pr*, whereas ${\tilde{s}}_{pr}^{2}$ refers to the posterior sample variance for *pr*. The statistic to compute the contrast estimator for the probe *pr* is formulated as follows: ${\hat{\beta }}_{pr}|\sigma_{pr}^{2}∼N\left(\beta_{pr},\sigma_{pr}^{2}\right)$ . Here, *N* represents the normal distribution. The statistic to estimate the posterior sample variance for *pr* is formulated as follows:

$${\tilde{s}}_{pr}^{2}=\frac{d_{0}s_{0}^{2}+d_{pr}s_{pr}^{2}}{d_{0}+d_{pr}},$$ (2)

where *d*_0_ (<∞) signifies the prior degrees of freedom, and $s_{0}^{2}$ denotes the variance. In addition, *d*_*pr*_ (>0) symbolizes the experimental degrees of freedom of *pr*, and $s_{pr}^{2}$ denotes the sample variance of *pr*. The significance of the level of the *p*-value is then determined from ${\tilde{s}}_{pr}^{2}$ with the help of the cumulative distribution function (cdf). If the *p*-value of the feature is less than the standard cutoff of 0.05, the feature is defined as statistically significant. The filtered differentially expressed features are then ordered according to the *p*-values. Notably, if any gene corresponds to more than one probe (feature), the probe with the lowest *p*-value will be selected to represent the gene, and the rest of the probes for the gene are simply ignored. We apply the same approach to each layer of the molecular profile, and then, we perform the combination of the significant non-redundant features (genes/probes/copy number variation, etc.) from all layers (let, *UF*).

### 2.2 Fusion by matrix factorization

Let *o*_*i*_ and *o*_*j*_ denote two object types, namely, gene expression and DNA methylation, in all resulted features *UF*. The number of genes is N, while each gene is denoted by *n*_*i*_, where i = 1, 2,…, N. There are M number of DNA methylation samples, while each sample is termed as *m*_*j*_, where j = 1, 2, …, M. In addition, there is a *P* set consisting of *p* types of profiles from the multi-omics datasets. The input to this implemented variant of the 3-*FPNMF* model is R , which is a relational block matrix shown as follows:

$$R=\left[\begin{array}{cccc} {}* & R_{12} & \ldots & R_{1p}\\ R_{21} & * & \ldots & R_{2p}\\ \vdots & \vdots & \ddots & \vdots \\ R_{p1} & R_{p2} & \ldots & * \end{array}\right].$$ (3)

Here, ∗ denotes that similar object relationships are not considered in this approach. *R*_*ij*_ denotes the relationship between *o*_*i*_th and *o*_*j*_th object types. The respective correlation of the *x*th object of type *o*_*i*_ (e.g., gene) and the *y*th object of type *o*_*j*_ (e.g., sample) is represented as $R_{o_{i}o_{j}}\left(x,y\right)$ . In this implementation, we have experimented with six object types, as described later.

For each object type from each profile, there is a constraint in the input constraint block diagonal matrix, as shown in the following expression:

$$\tau^{P}=Diag\left(\tau^{1},\tau^{2},\ldots ,\tau^{p}\right).$$ (4)

The relational block matrix R is tri-factorized into matrix factors *G* and *S* (Žitnik and Zupan, 2014), which is shown as follows:

$$G=Diag\left(G_{n_{1}\times m_{1}}^{1},G_{n_{2}\times m_{2}}^{2},\ldots ,G_{n_{p}\times m_{p}}^{p}\right),$$ (5)

$$S=\left[\begin{array}{cccc} {}* & S_{12}^{r_{1}\times r_{2}} & \ldots & S_{1p}^{r_{1}\times r_{p}}\\ S_{21}^{r_{2}\times r_{1}} & * & \ldots & S_{2p}^{r_{2}\times r_{p}}\\ \vdots & \vdots & \ddots & \vdots \\ S_{p1}^{r_{p}\times r_{1}} & S_{p2}^{r_{p}\times r_{2}} & \ldots & * \end{array}\right].$$ (6)

Here, *r* denotes rank factorization to the object type *o*_*p*_ inferenced by the 3-*FPNMF* model. The factor *S* denotes the block relation between object types *o*_*i*_ and *o*_*j*_. The factor $G_{o_{i}}$ reconstructs relations specifically to the object type *o*_*i*_.

Thus, each relation matrix $R_{o_{i}o_{j}}$ obtains matrix factorization as $G_{o_{i}}S_{o_{i}o_{j}}G_{o_{j}}^{T}$ . In a simplified way, this relational block 3-*FPNMF* model is shown as follows:

$$\left[\begin{array}{cccc} {}* & G_{o_{1}}S_{o_{1}o_{2}}G_{o_{2}}^{T} & \ldots & G_{o_{1}}S_{o_{1}o_{p}}G_{o_{p}}^{T}\\ G_{o_{2}}S_{o_{2}o_{1}}G_{o_{1}}^{T} & * & \ldots & G_{o_{2}}S_{o_{2}o_{p}}G_{o_{p}}^{T}\\ \vdots & \vdots & \ddots & \vdots \\ G_{o_{p}}S_{o_{p}o_{1}}G_{o_{1}}^{T} & G_{o_{p}}S_{o_{p}o_{2}}G_{o_{2}}^{T} & \ldots & * \end{array}\right].$$ (7)

The objective function of this tri-factor penalized matrix decomposition (*PMD*) model is to minimize the distance between the input block relational matrix R and its 3-*FPNMF* system adhering to the constraint matrix *τ* ^*P*^, which is shown as follows:

$$\begin{array}{ccc} \underset{G\geq 0}{\min}j\left(R:G,S\right)=\underset{R_{o_{i}o_{j}}\in R}{\sum }\|R_{o_{i}o_{j}}-G_{o_{i}}S_{o_{i}o_{j}}G_{o_{j}}^{T}{\|}^{2} & & \\ +\overset{P}{\underset{p=1}{\sum }}tr\left(G^{T}\tau^{p}G\right) & & . \end{array}$$ (8)

**FIGURE 1:**
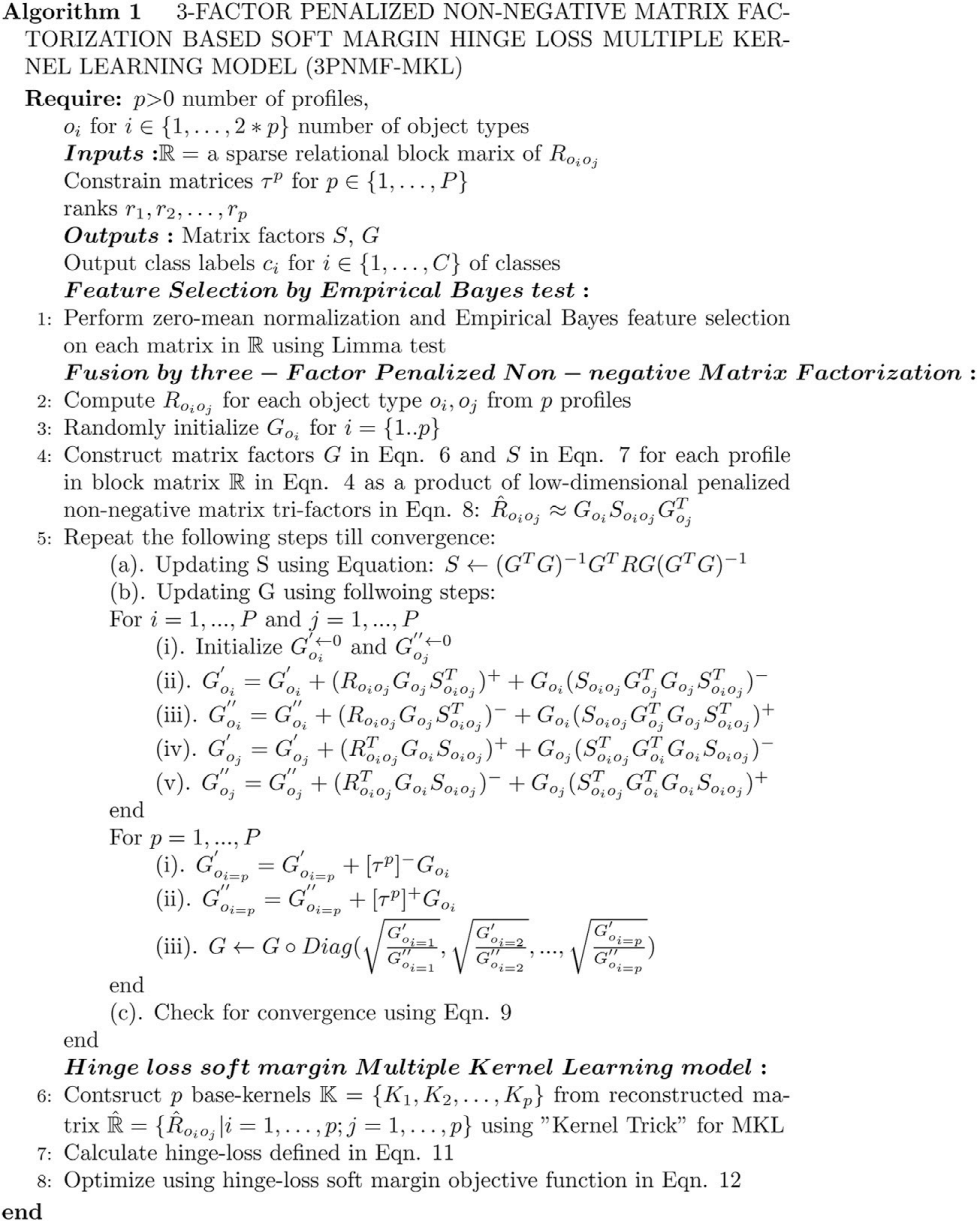
Algorithm of the proposed 3PNMF-MK model.

Here, ‖.‖ denotes the Frobenius norm, and *tr* (.) denotes the trace. Our sparse implementation for this 3-*FPNMF* model reduces the missing relational matrix problem with zero values. Our model is more suitable for real-life heterogeneous datasets with missing values, which differs from those of Žitnik and Zupan (2014) in its non-negative sparse implementation. Our proposed 3*FPNMF* − *MKL* model is shown briefly in Figure 1, while a detailed flowchart is represented in Supplementary Figure S1.

### 2.3 Multiple kernel learning

Next, we introduce the multiple Kernel Learning (MKL) algorithm (Xu et al., 2013) with the hinge loss soft margin, in which the classifier and the kernel combination coefficients are optimized by solving the hinge loss soft margin MKL problem.

After using the 3-*FPNMF* model in the first phase, the approximate sparse relation matrix ${\hat{R}}_{o_{i}o_{j}}$ for target object type pairs *o*_*i*_ and *o*_*j*_ is reconstructed as

$${\hat{R}}_{o_{i}o_{j}}=G_{o_{i}}S_{o_{i}o_{j}}G_{o_{j}}^{T}.$$ (9)

Then, to develop kernel fusion, the resulting kernel matrices are generated using the “Kernel Trick”: $K\left(o_{i},o_{j}\right)={\hat{R}}_{o_{i}o_{j}}.{\hat{R}}_{o_{i}o_{j}}^{T}$ . The kernels are further normalized and smoothed using 2-dimensional linear filters.

Given *p* base-kernels $K=\left\{K_{1},K_{2},\ldots ,K_{p}\right\}$ developed from the reconstructed relational block matrix $\hat{R}=\left\{{\hat{R}}_{o_{i}o_{j}}|i=1,\ldots ,p;j=1,\ldots ,p\right\}$ , kernel slack variables for the kernel $K_{p}\in K$ are defined as the difference between the target margin *θ* and the SVM dual objective function

$$DSVM\left(K_{p},\alpha \right)$$

$$=\underset{\alpha \in R^{N}}{\max}\overset{N}{\underset{n=1}{\sum }}\alpha_{n}-\frac{1}{2}\overset{N}{\underset{m=1}{\sum }}\overset{N}{\underset{n=1}{\sum }}\alpha_{n}\alpha_{m}y_{n}y_{m}K_{p}\left(x_{n},x_{m}\right)$$

subject to $\sum_{n=1}^{N}y_{n}\alpha_{n}=0,\alpha_{n}\geq 0,\forall n$ . Then, the slack variable is *ζ*_*p*_ = *θ* − *DSVM*(*K*_*p*_, *α*), and the hinge loss is shown as follows:

$$z_{p}=\ell \left(\zeta_{p}\right)=\max\left(0,\zeta_{p}\right).$$ (10)

Therefore, the objective function for this hinge loss soft margin MKL algorithm becomes

$$\underset{\theta ,\alpha \in Dom\left(\alpha \right),\zeta_{p}}{\min}-\theta +\pi \overset{P}{\underset{p=1}{\sum }}\zeta_{p}.$$ (11)

subject to *DSVM*(*K*_*p*_, *α*) ≥*θ* − *ζ*_*p*_, *ζ*_*p*_ ≥ 0, *p* = 1, *…*, *P*.

**FIGURE 2:**
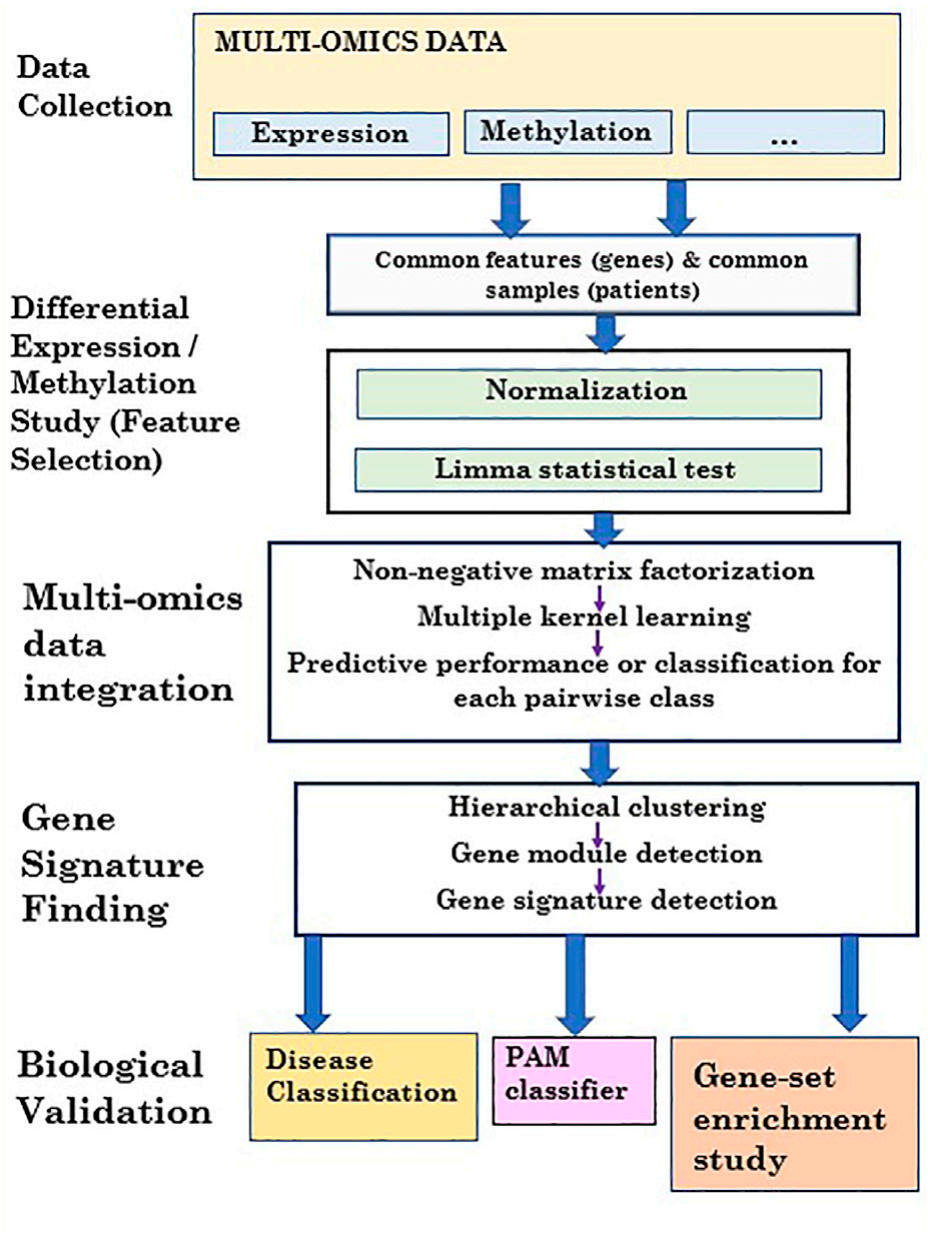
Flowchart of the proposed 3PNMF-MKL framework.

The objective of the aforementioned hinge loss soft margin MKL is to maximize the margin *θ* while considering the “errors” from the given *P*-based kernels. The parameter *π* balances the contribution of the loss term represented by slack variables *ζ*_*p*_ and the margin *θ*. *π* should be in the range {*π*|*π* ≥ 1/*P*}. Otherwise, there is no solution to the proposed problem. Our proposed framework for gene signature detection from heterogeneous data sources using the 3*FPNMF* − *MKL* model is depicted in Figure 2.

### 2.4 Determining best combination of class labels using non-matrix factorization and AUC

In biological datasets such as TCGA, the clinical data are made available. This includes patient sample groups, biological subtypes, drug treatment, and survival/prognosis information. In our current study, we obtain accuracies for different combinations of class labels using the non-matrix factorization technique for the case where there were more than two class labels or subtypes. Among them, the combination of class labels, which produces the highest area under curve (AUC), is chosen for the next step (i.e., module detection). Say, q is the specific combination of class labels, which produces the highest AUC. Find *q* = {*∃i*, *∃j*}|{*∃a*, *∃b*, *∃k*} such that

$$AUC_{q}=\arg\max\left(\forall_{i,j}AUC_{cl_{i}cl_{j}^{\prime }},\forall_{a,b,k}AUC_{cl_{a}cl_{bk}^{\prime }}\right),$$ (12)

where *cl* denotes the left part of the group combination, *cl*′ signifies the right part of any sample group combination, and *i* ∈ {1, 2, …, (*m* − 1)}, *j* ∈ {(*i* + 1), (*i* + 2), …, *m*}, *a* ∈ {1, 2, …, *m*}, *b* ∈ {1, 2, …, *m*} & *b* ≠ *a*, *k* ∈ {, 2, …, *m*}, and *k* ≠ *a* and *k* ≠ *b*.

### 2.5 Feature clustering and module detection

After selecting the right class-label combination, we extracted the sub-gene expression data consisting of only the selected class labels and then used them for gene module detection and signature identification.

In our procedure, we first evaluated the power of the soft thresholding, which was then applied to evaluate the adjacency matrix using Pearson’s correlation. The topological overlap matrix (TOM) similarity score (Ravasz et al., 2002) was computed from the employed adjacency matrix. The TOM score between two nodes (say, *i* and *j*) symbolized as *TOM*(*i*, *j*) is defined as follows:

$$TOM\left(i,j\right)=\left\{\begin{array}{cc} \frac{\underset{v\neq i,j}{\sum }X\left(i,v\right)X\left(j,v\right)+X\left(i,j\right)}{\min\left\{\underset{v\neq i}{\sum }X\left(i,v\right),\underset{v\neq j}{\sum }X\left(j,v\right)\right\}-X\left(i,j\right)+1},\; & \text{if}i\neq j,\\ 1,\; & \text{if i}=\text{j}, \end{array}\right.$$ (13)

where *X* denotes the corresponding adjacency matrix containing Boolean entries. The entry of 1 indicates that the corresponding two nodes share the same connection (i.e., direct connection), while the entry of 0 signifies that no direct connection exists between them.

After obtaining the TOM score, we computed the distance/dissimilarity value between genes (*i* and *j*) denoted by *dissTOM*(*i*, *j*),which is shown as follows: *dissTOM*(*i*, *j*) = 1 − *TOM*(*i*, *j*). We conducted average linkage clustering on the multi-omics dissimilarity matrix *dissTOM* *via* considering all potential pairs of genes/features. Finally, the dynamic tree cut technique (Langfelder et al., 2008) was applied on the clustering dendrogram to determine the gene modules. In order to evaluate the quality of the aforementioned clustering, we calculated different cluster validity index measures, *viz.*, cluster coefficient, heterogeneity, Dunn Index, maximum adjacency ratio, centralization, silhouette width, and scaled connectivity.

### 2.6 Expression signature detection and classifier models

After finding the gene modules, we estimated Pearson’s correlation coefficient (PCC) between each gene pair within the resulted modules. For each module, the mean of the correlations for each gene pair within that particular module was obtained. The module with the maximum mean correlation coefficient was elected as a gene signature. Notably, genes with the elected gene signature are differentially expressed between case and control samples. In order to validate the classification performance of the employed gene signature, we utilized the Prediction Analysis of Microarrays (PAM) classifier with 10-fold cross-validation (CV) on the expression sub-data to classify the underlying class labels. The entire procedure was then repeated ten times. Moreover, we calculated the average scores of several classification performance metrics such as sensitivity, specificity, precision, accuracy, and AUC, individually.

### 2.7 Functional annotation analysis

We carried out KEGG pathway and Gene Ontology (GO) analyses using the Enrichr database (Chen et al., 2013). Notably, GO terms can be categorized into three kinds, *viz.*, biological process (BP), cellular component (CC), and molecular function (MF). Those significant pathways/GO terms with an adjusted *p*-value less than 0.05 were identified. Meanwhile, literature research studies were also performed to identify disease-related pathways/GO terms.

## 3 Results

### 3.1 Data sources

For our experiment, TCGA acute myeloid leukemia (LAML) multi-omics dataset (https://xenabrowser.net/datapages/?cohort=GDC%20TCGA%20Acute%20Myeloid%20Leukemia%20(LAML)&removeHub=https%3A%2F%2Fxena.treehouse.gi.ucsc.edu%3A443) contained six heterogeneous profiles such as the gene expression (IlluminaGA) profile, DNA methylation (Illumina Methylation 27k) profile, exon expression (IlluminaGA) profile, miRNA profile, pathway activity (Paradigm IPLs) profile, and copy number (GISTIC2) profile. Initially, the gene expression profile included 179 samples and 20,113 genes. For the methylation profile, there are 194 samples and 27,578 methylation probes. Particularly, for the methylation profile, many genes are profiled with more than one probe. In the exon expression profile, there are a total of 219,296 chromosome ids and 179 samples. Here, many genes are connected with more than one chromosome id. The miRNA profile contains 705 miRNAs and 188 samples. The pathway activity profile has 7,203 genes and 173 samples, while the copy number profile consists of 24,776 genes and 191 samples. There are three categories of samples (i.e., class labels) for the LAML multi-omics dataset: i) favorable, ii) intermediate (also called normal), and iii) poor. Specifically, every profile consists of 161 commonly shared LAML samples. Among them, 31 samples belong to the first category, 96 samples are in the second category, and the rest of the samples (= 34) are in the third category. In addition, there are 1,501 uniquely matched genes among the five profiles [i.e., gene expression, DNA methylation, exon expression, pathway activity, and copy number variation (GISTIC2) profiles].

### 3.2 Statistical validation

First, we selected the sub-data, which contain commonly shared samples (i.e., 161) and genes (i.e., 1,501) for each of the five profiles (i.e., gene expression, DNA methylation, exon expression, pathway activity, and copy number variation profiles). Many matched genes are connected with more than one probe (or chromosome id) for each profile. In the case of the miRNA profile, we started to work with the matched samples (*n* = 161) and all of its miRNAs (*n* = 705). The empirical Bayes test is performed by limma software on each gene probe or chromosome id for each of the five profiles (i.e., gene expression, DNA methylation, exon expression, pathway activity, and copy number variation profiles) across all the three classes (viz., favorable, intermediate, and poor).

Notably, since there are three classes/groups of samples, here, limma is initially performed between each group pair (i.e., i) favorable vs. intermediate, ii) intermediate vs. poor, and finally iii) favorable vs. poor), then an F-statistics is computed, and finally, the respective *p*-value is generated from the F-statistics. After the test, for every gene, we only selected the probe or chromosome id with the lowest *p*-value achieved among all the probes or chromosome ids connected with that gene. As a result, we obtained 728, 272, 1,100, 265, and 904 significant genes for the gene expression, methylation, exon expression, pathway activity, and copy number profiles, respectively. Thereafter, we took the combination of all the significant gene sets, which led to a molecular set of a total of 1,388 genes. Furthermore, the same significance test was applied on each miRNA of the miRNA profile across all the three classes (*viz.*, favorable, intermediate, and poor) as well. We obtained a total of 229 significant miRNAs.

### 3.3 Expression signature detection and classification

**FIGURE 3:**
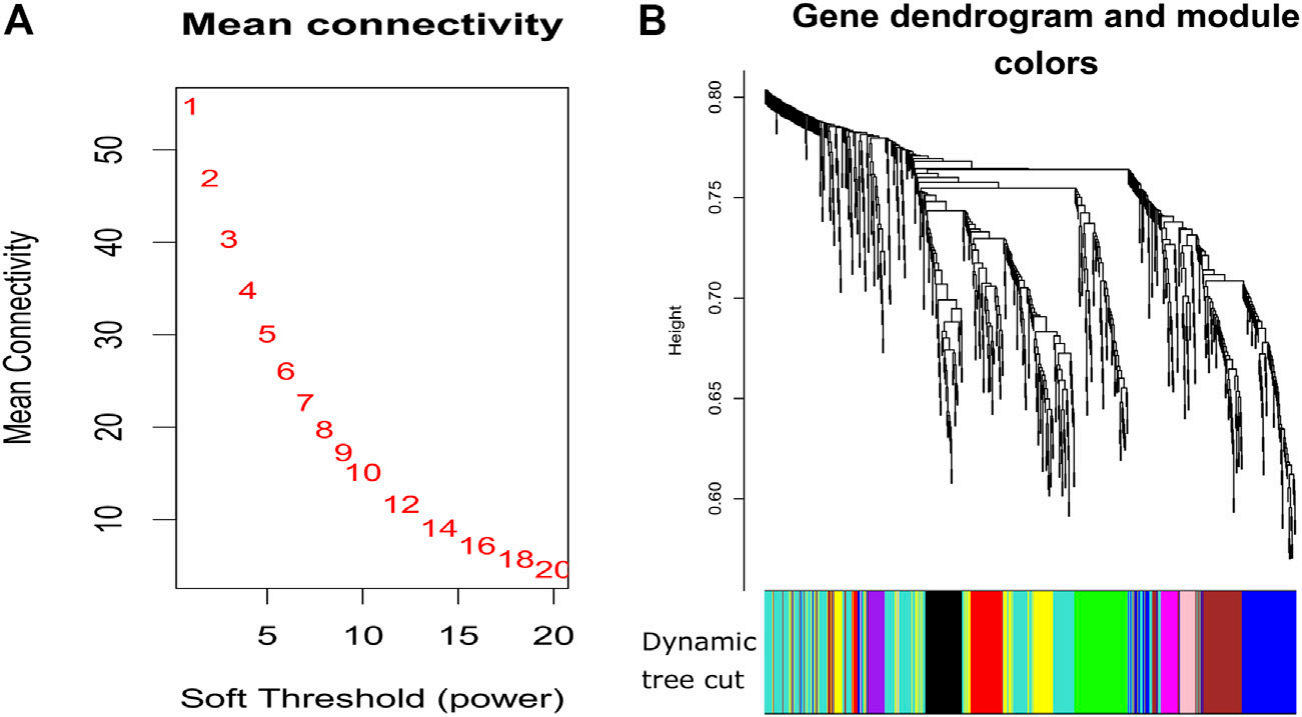
Plots for soft thresholding and dendrogram for our proposed method. **(A)** Power computing for soft thresholding and **(B)** dendrogram plots with dynamic tree cut.

**FIGURE 4:**
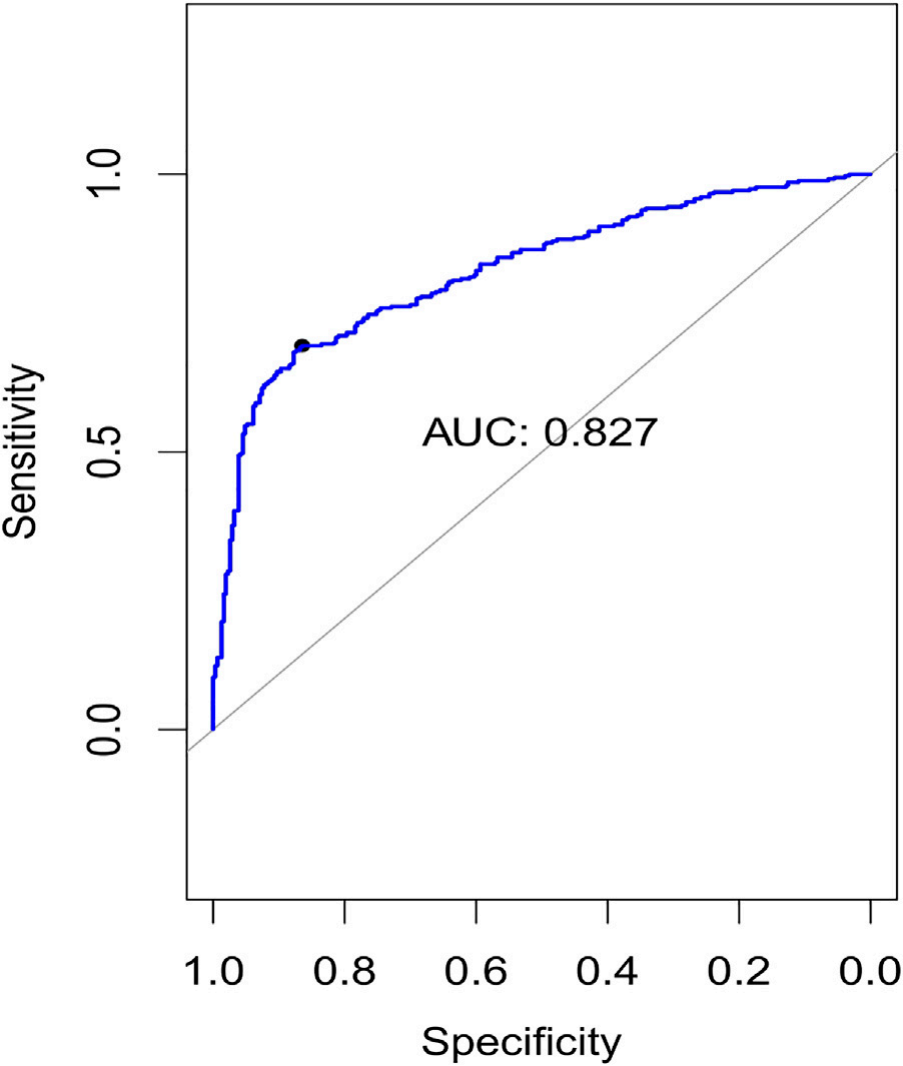
Plots of the area under curve (AUC) for 10-fold cross-validation.

Using the non-matrix factorization technique, we obtained accuracies for different combinations of class labels such as i) Class 1 (favorite) vs. Class 2 (intermediate), ii) Class 1 vs. Class 3 (Poor), iii) Class 1 vs. classes 2 and 3, iv) Class 2 vs. Class 3, v) Class 2 vs. classes 1 and 3, and vi) Class 3 vs. Classes 1 and 2 (as depicted in Table 1). Among them, the second combination, i.e., Class 1 vs. Class 3 produced the highest area under curve (AUC = 0.7713). Hence, we selected the combination for gene signature discovery since other combinations did not produce better AUC scores. After obtaining right combinations of class labels, we first evaluated the power (=1) for soft thresholding (illustrated in Figure 3A), which was then applied to estimate the adjacency matrix through Pearson’s correlation score. Then, the TOM score and distance matrix were computed. To determine gene modules, we applied average linkage clustering and dynamic tree cut methodologies. As a result, we generated a total of 10 gene modules. The numbers of participating differentially expressed genes (DEGs) for these 10 gene modules (represented by black, blue, brown, green, magenta, pink, purple, red, turquoise, and yellow colors) were 50, 99, 90, 74, 23, 25, 22, 51, 214, and 80, respectively. The dendrogram is represented in Figure 3B. The corresponding cluster validity indices in that module detection are illustrated in Table 2. The Average silhouette width plot generated during clustering is represented in Supplementary Figure S2. PCC was calculated between each gene pair within each module. The mean correlation scores of the 10 modules (depicted by blue, green, turquoise, magenta, brown, red, yellow, black, purple, and pink colors) were 0.0268, 0.2562, 0.0321, 0.3914, 0.1143, 0.0215, 0.0570, 0.4029, 0.3455, and 0.1605, respectively. The black module had the highest mean correlation coefficient score (= 0.4029 in Table 3). Thus, it was selected as the gene signature. The resultant gene signature contained 50 DEGs (see Table 3). To verify the classification performance of the resultant signature, we applied the PAM classifier through the 10-fold cross-validation (CV) on all the features and samples of signature data in order to classify the groups (favorite and poor). The entire procedure was then repeated 10 times. In the experiment, the mean sensitivity, mean specificity, mean precision, mean accuracy, and mean AUC were 69.12%, 84.19%, 82.79%, 76.31, and 0.8273, respectively (see Figure 4; Table 4). Based on the gene set enrichment analysis on the 50 genes of the signature using the Enrichr web database, we extracted significant KEGG pathway and Gene Ontology (GO) terms. Among the KEGG pathways, the Rap1 signaling pathway (hsa04015) is the most significant pathway (adjusted *p*-value = 7.497 × 10^−06^) that contains eight genes (viz., *EFNA1*, *GNAO1*, *TIAM1*, *CSF1*, *ITGB3*, *ITGA2B*, *THBS1*, and *MAPK13*). Second, the most significant pathway is the PI3K-Akt signaling pathway (hsa04151) with an adjusted *p*-value of 1.128 × 10^−05^, which consists of nine genes (viz., *EFNA1, CSF1, ITGB3, ITGA2B, IL2RB, FASLG, TP53, THBS1*, and *EPOR*). The following eight pathways are the cytokine–cytokine receptor interaction (hsa04060) (adj. *p*-value = 1.437 × 10^−05^), inflammatory bowel disease (IBD) (hsa05321) (adj. *p*-value = 2.1E-05), proteoglycans in cancer (hsa05205) (adj. *p*-value = 2.1 × 10^−05^), hematopoietic cell lineage (hsa04640) (adj. *p*-value = 6.752 × 10^−05^), T-cell receptor signaling pathway (hsa04660) (adj. *p*-value = 1 × 10^−4^), TNF signaling pathway (hsa04668) (adj. *p*-value = 2 × 10^−4^), osteoclast differentiation (hsa04380) (adj. *p*-value = 3 × 10^−4^), and Ras signaling pathway (hsa04014) (adj. *p*-value = 3 × 10^−4^) (also see Table 5). Among the significant GO:BP terms, the positive regulation of cellular metabolic processes (GO:0031325) (adjusted *p*-value = 8.02947 × 10^−05^) was ranked as the most significant, which contains six genes (*EDN1*, *CSF1*, *CCL5*, *GATA3*, *THBS1*, and *TP53*). The second most significant GO term is the regulation of inflammatory responses (GO:0050727) with an adjusted *p*-value of 8.029 × 10^−05^. This term consists of seven genes (*CCL5, CCL4, RORA, GATA3, ETS1, BIRC3*, and *MAPK13*) (Table 5). Among the significant GO:CC terms, the platelet alpha granule (GO:0031091) (adjusted *p*-value = 4 × 10^−3^) contains four genes (viz., *ITGB3, ITGA2B, A2M*, and *THBS1*), while among the GO:MF terms, the core promoter binding factor (GO:0001047) (adjusted *p*-value = 8 × 10^−4^) contains five genes (*viz.*, *RORA, GATA3, GATA1, TP53*, and *ARNTL*). For details of the top significant pathways and GO terms, see Table 5.

**TABLE 1:** Predictive performance of classification for each pairwise class using the proposed method in LAML multi-omics data, where classes 1, 2, and 3 denote “favorable,” “intermediate/normal,” and “poor,” respectively.

	Sensitivity	Specificity	Precision (PPV)	Negative predictive value	Accuracy	AUC
Class 1 vs. Class 2	0.5161	0.6907	0.3478	0.8171	0.6484	0.6202
Class 1 vs. Class 3	0.5484	0.8235	0.7391	0.6667	0.6923	0.7713
Class 1 vs. classes 2 and 3	0.5385	0.3871	0.7865	0.1667	0.5093	0.4608
Class 2 vs. Class 3	0.6289	0.5	0.7821	0.3208	0.5954	0.5215
Class 2 vs. classes 1 and 3	0.5	0.5052	0.4	0.6049	0.5031	0.4863
Class 3 vs. classes 1 and 2	0.5547	0.4848	0.8068	0.2192	0.5404	0.5528
Max	0.6289	0.8235	0.8068	0.8171	0.6923	0.7713

**TABLE 2:** Cluster Validity Index measures of our experiment.

Cluster Validity Index	Score
Dunn Index	0.6461
Average scaled connectivity	0.6834
Silhouette width	−0.0012
Average cluster coefficient	0.2390
Average maximum adjacency ratio	0.2386
Density	0.2327
Centralization	0.1081
Heterogeneity	0.1143

**TABLE 3:** Feature (gene) names and average (avg.) Pearson’s correlation coefficient (PCC) for the pairwise manner within the TCGA LAML signature.

Measure	Value/description
# Features	50
Gene symbols	*HK2*, *CHRDL1*, *EFNA1*, *ARNTL*, *EIF4A1*, *MS4A2*, *BMP2*, *FHL2*, *SH2D2A*, *CSF1*, *KLRG1*, *ITGB3*, *SH3BP5*, *CCL4*, *RORA*, *CAMK2D*, *BIRC3*, *TP53*, *S1PR5*, *GNAZ*, *EPOR*, *TBX21*, *GATA3*, *TIAM1*, *IL2RB*, *LRIG1*, *GRAP2*, *PLEKHA1*, *THBS1*, *MAF*, *IL18RAP*, *EDN1*, *ETS1*, *GATA1*, *ITGA2B*, *A2M*, *LCK*, *MAPK13*, *GZMB*, *PTGDR*, *MYBL1*, *RASGRP1*, *ARG1*, *PKLR*, *GNAO1*, *PRF1*, *CD8A*, *FASLG*, *ABCG2*, and *CCL5*
Average PCC	0.403

**TABLE 4:** Classification metrics for our experiment.

Evaluation metric	Average score (std)
Precision	0.8279 (±0.027)
Sensitivity	0.6912 (±0.025)
Specificity	0.8419 (±0.028)
Accuracy	0.7631 (±0.0208)
AUC	0.8273

**TABLE 5:** Top five significant KEGG pathways and Gene Ontology (GO) terms* for the gene set belonging to the LAML signature.

KEGG pathway name	Gene symbol	Z-score	Adjusted *p*-value
Rap1 signaling pathway (hsa04015)	*EFNA1*, *GNAO1*, *TIAM1*, *CSF1*, *ITGB3, ITGA2B*, *THBS1*, and *MAPK13*	−1.961	7.497 × 10−06
PI3K-Akt signaling pathway (hsa04151)	*EFNA1*, *CSF1*, *ITGB3*, *ITGA2B*, *IL2RB*, *FASLG*, *TP53*, *THBS1,* and *EPOR*	−2.041	1.128 × 10−05
Cytokine–cytokine receptor interaction (hsa04060)	*BMP2*, *IL18RAP*, *CSF1*, *CCL5*, *IL2RB*, *CCL4*, *FASLG*, and *EPOR*	−1.829	1.437 × 10−05
Inflammatory bowel disease (IBD) (hsa05321)	*MAF*, *IL18RAP*, *TBX21*, *RORA*, and *GATA3*	−1.858	2.1 × 10−05
Proteoglycans in cancer (hsa05205)	*TIAM1*, *CAMK2D*, *ITGB3*, *FASLG*, *TP53*, *THBS1*, and *MAPK13*	−1.910	2.1 × 10−05
Positive regulation of the cellular metabolic process (GO:BP: GO:0031325)	*EDN1*, *CSF1*, *CCL5*, *GATA3*, *THBS1*, and *TP53*	−1.551	8.029 × 10−05
Regulation of inflammatory response (GO:BP: GO:0050727)	*CCL5*, *CCL4*, *RORA*, *GATA3*, *ETS1*, *BIRC3*, and *MAPK13*	−1.029	8.029 × 10−05
Positive regulation of gene expression (GO:BP: GO:0010628)	*BMP2*, *CSF1*, *TBX21*, *FHL2*, *RORA*, *GATA3*, *ETS1*, *GATA1*, *MYBL1*, *THBS1*, *TP53*, and *ARNTL*	−1.668	8.029 × 10−05
Cytokine-mediated signaling pathway (GO:BP: GO:0019221)	*CAMK2D*, *IL18RAP*, *CSF1*, *CCL5*, *CCL4*, *IL2RB*, *FASLG*, *RORA*, *GATA3*, *TP53*, and *BIRC3*	−1.343	8.029 × 10−05
Positive regulation of nucleic acid-templated transcription (GO:BP: GO:1903508)	*BMP2*, *TBX21*, *FHL2*, *RORA*, *GATA3*, *ETS1*, *GATA1*, *MYBL1*, *TP53*, and *ARNTL*	−2.001	8.029 × 10−05
Platelet alpha-granule (GO-CC: GO:0031091)	*ITGB3*, *ITGA2B*, *A2M*, and *THBS1*	−1.639	4 × 10−3
Platelet alpha-granule membrane (GO-CC: GO:0031092)	*ITGB3* and *ITGA2B*	−2.148	0.023
Core promoter binding (GO-MF: GO:0001047)	*RORA*, *GATA3*, *GATA1*, *TP53*, and *ARNTL*	−1.279	8 × 10−4
Core promoter sequence-specific DNA binding (GO-MF: GO:0001046)	*RORA*, *GATA3*, *GATA1*, and *TP53*	−1.295	1.9 × 10−3
Transcription regulatory region DNA binding (GO-MF: GO:0044212)	*TBX21*, *RORA*, *GATA3*, *GATA1*, *MYBL1*, *TP53*, and *ARNTL*	−1.322	1.9 × 10−3
Cytokine activity (GO-MF: GO:0005125)	*BMP2*, *EDN1*, *CSF1*, *CCL5*, and *CCL4*	−1.224	2 × 10−3
Transcription factor activity and RNA polymerase II core promoter proximal region sequence-specific binding (GO-MF: GO:0000982)	*GATA3*, *ETS1*, *GATA1*, *MYBL1*, *TP53*, and *ARNTL*	−1.604	2.2 × 10−3

*Gene Ontology (GO) has three domains: biological process (BP), cellular component (CC), and molecular function (MF).

## 4 Discussion

Multi-view data integration and gene signature detection are currently the most challenging tasks for biomedical researchers. As different datasets contain different characteristics, integration of data from multi-platforms with significant feature reduction and gene module detection will give a more comprehensive view of how biology unravels at a granular level. Therefore, we introduced the novel approach of multi-platform data integration technique, 3PNMF-MKL, for multi-platform data integration and gene signature detection. This approach applies the integrated utilization of statistical methods, data fusion through three-factor penalized non-negative matrix factorization, and soft margin hinge loss-based multiple kernel learning. We then tested our approach using TCGA LAML multi-omics dataset, which contains five different profiles (viz., gene expression, DNA methylation, exon expression, pathway activity, and copy number). Overall, our algorithm provides excellent AUC (= 0.827) for classifying the class labels for the underlying features (genes) within the chosen gene signature. Furthermore, we performed a functional analysis using the KEGG pathway and Gene Ontology database to interpret those identified relevant feature genes. Collectively, our novel approach is applicable to any kind of multi-modal datasets.

Our proposed method 3PNMF-MKL includes data integration employed by means of differential expression/methylation analysis using limma, non-negative matrix factorization, and soft margin hinge loss, as well as gene signature detection together. 3PNMF-MKL employs the application of best gene module discovery with the help of dynamic linkage clustering, dynamic tree cut, and correlation analysis to achieve the use of best gene module discovery (in terms of gene signature discovery) . So far, there are many state-of-the-art methods available regarding data integration (Yang and Michailidis, 2016; Ray et al., 2017) and gene signature discovery (Cun and Frohlich, 2012; (Zhang and Xiao, 2020), but very few existing methods are recently available where data integration and gene signature detection work together in the same framework (Fujita et al., 2018). We, here, compared our proposed method 3PNMF-MKL with the existing method (Zhang and Xiao, 2020) used for TCGA acute myeloid leukemia dataset. In our proposed method, we obtained a 50-gene signature generated after analyzing multi-omics data integration where the other method (Zhang and Xiao, 2020) produced an eight-gene signature from analyzing the only gene expression data not by multi-omics data integration. Also, we obtained 0.87 as the training set’s 1-year AUC and 0.72 as the test set’s 1-year AUC in the signature survival study (by cox regression), while the other method obtained 0.86 as the training set’s 1-year AUC and 0.69 as the test set’s 1-year AUC for the gene expression data. Therefore, in all perspectives, our signatures are stronger than the other.

## 5 Conclusion and future directions

No method, which deals with data integration non-matrix factorization, soft margin hinge loss, and gene signature together, exists in the field of bioinformatics, whereas our work is concerned with the process of integration of multi-omics data employing multi-dimensional schemes such as differential expression/methylation analysis using limma, non-negative matrix factorization, soft margin hinge loss, and gene signature detection through the use of best gene module discovery using dynamic linkage clustering, dynamic tree cut method, and correlation analysis, respectively. The achievement of a high classification accuracy of 0.8273 also represents superior performance for our proposed algorithm. In addition, our method outperformed the state-of-the-art methods in terms of computing AUC. Expansion of our current approach with a deep learning strategy to tackle the integrative problem at a single-cell level is our future directive. In future work, we will collaborate with a wet laboratory to validate our experimental results in order to make it more promising.

## Data Availability

The original contributions presented in the study are included in the article/Supplementary Material, further inquiries can be directed to the corresponding authors.

## Supplementary material

SUPPLEMENTARY FIGURE S1 Detailed flowchart of the proposed 3PNMF-MKL framework.

SUPPLEMENTARY FIGURE S2 Average silhouette width during clustering.
