# Supplementary figures and images for: 3PNMF-MKL: A non-negative matrix factorization-based multiple kernel learning method for multi-modal data integration and its application to gene signature detection
Source: Front Genet. 2023 Feb 14;14:1095330. doi: 10.3389/fgene.2023.1095330 (PMC9971618; doi:10.3389/fgene.2023.1095330)

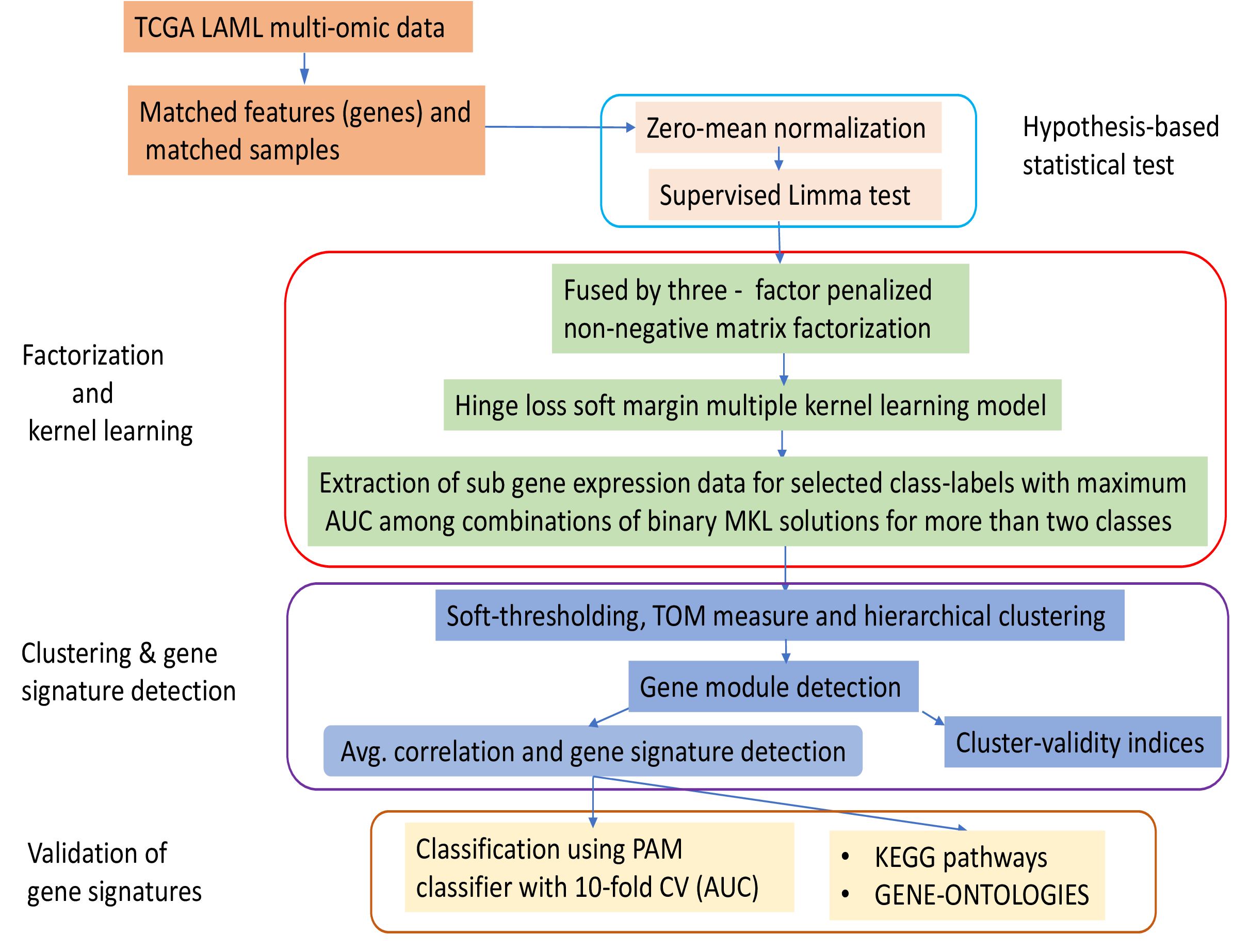

Supplement: Supplementary file 1 [file Image1.TIFF]

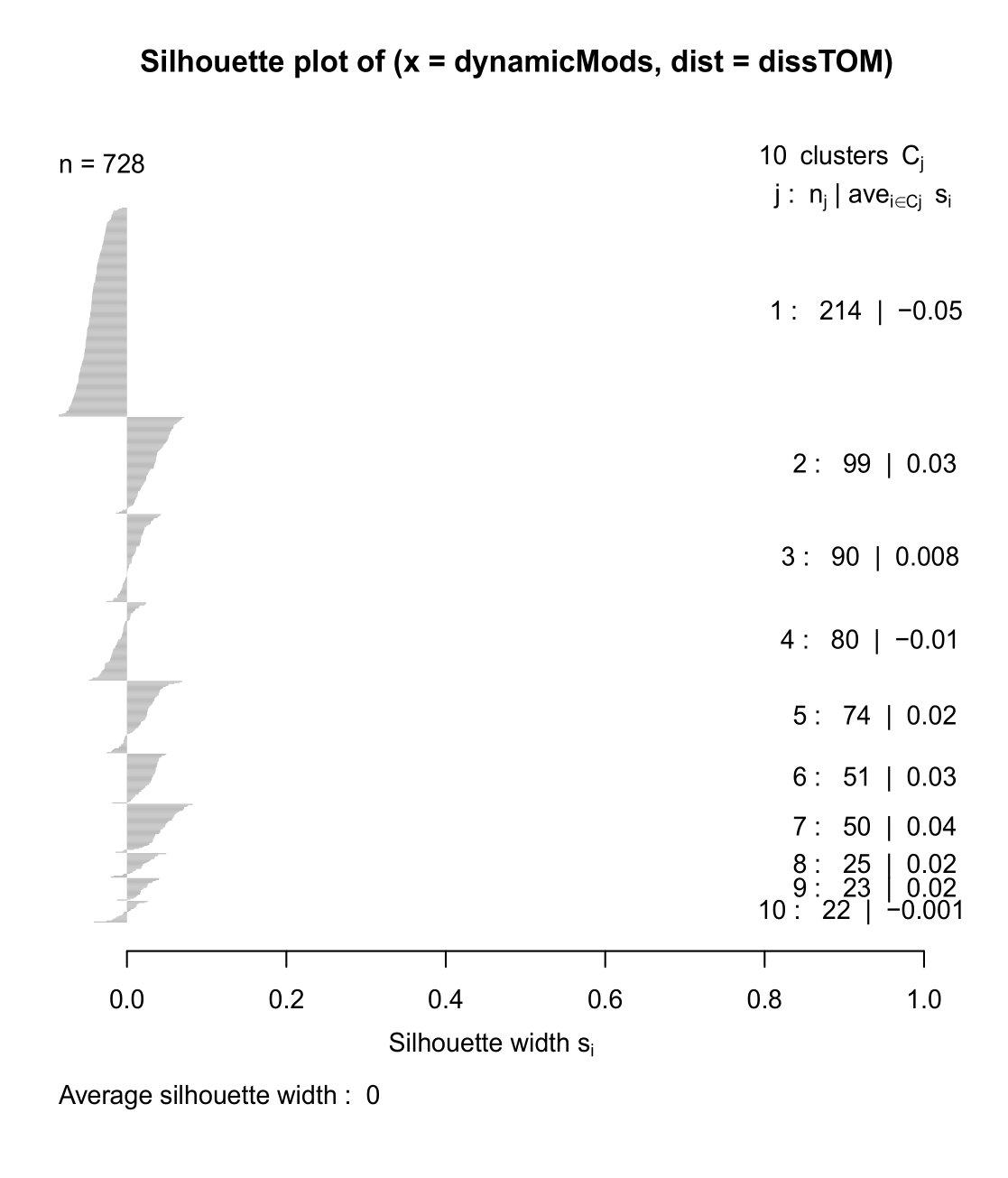

Supplement: Supplementary file 2 [file Image2.TIFF]
